# Conclusion: The Next Decade of Family Policy Research

**DOI:** 10.1007/978-3-030-54618-2_26

**Published:** 2020-10-10

**Authors:** Wim Van Lancker, Rense Nieuwenhuis

**Affiliations:** 1 Swedish Institute for Social Research (SOFI), Stockholm University, Stockholm, Sweden; 2 Centre for Sociological Research, University of Leuven, Leuven, Belgium; 3 Centre for Sociological Research, University of Leuven, Leuven, Belgium; 4 Swedish Institute for Social Research (SOFI), Stockholm University, Stockholm, Sweden

## Abstract

Based on the multilevel perspective on family policy research brought together in this handbook, this chapter highlights five major societal challenges for the future outlook and outcomes of family policies, and reflects on what the handbook teaches us about how to effectively address these challenges, as well as what there is yet to learn. The challenges pertain to the (1) levels of policy implementation, and in particular globalization and decentralization, (2) austerity and marketization, (3) economic inequality, (4) changing family relations, and (5) welfare states adapting to women’s empowered roles. The chapter concludes by examining what lessons were learned, and are yet to learn, regarding the capacity of family policies to cope with shocks of various kinds and to support families during extraordinary times.

Family policies are influenced, formulated, or implemented at levels that range from the supra-national, over the national and the sub-national to the level of organizations. At these different levels, family policies serve similar functions that include child income supports, childcare services, parental leaves, leaves to provide care to frail and elderly family members, and support similar goals that include improving children’s development and equal opportunities, promoting gender equality, regulating fertility, and stimulating productivity. This has been extensively documented in the chapters of this handbook.

The purpose of this final chapter is to look ahead. We introduce what we believe are five major societal challenges for the future outlook and outcomes of family policies, and reflect on what the handbook teaches us on how to effectively address these challenges, as well as what there is yet to learn. With the latter, we hope to contribute to setting the stage for the next decade of family policy research.

## Levels of Policy Implementation: Globalization and Decentralization

This handbook has been developed based on the premise that bringing together (insights into) different levels of family policy making provides a relevant and comprehensive understanding of family policies. Processes of globalization and regional integration (such as in the EU) on the one hand, and decentralization on the other, make that increasingly families are subject to policies at multiple levels of (potential) decision making. Family policies are increasingly available and studied across the globe; supra-national and international organizations are increasingly involved in analyzing, recommending, or implementing family policies, while regions and even cities are being delegated—or taking—responsibilities in family policy implementation. All at the same time, organizations play a crucial role in what access workers have to family policy arrangements, either limiting access to or supplementing publicly provided family policy provisions. The challenge for a research agenda that can successfully account for these developments is to develop a clear analytical and conceptual focus that goes beyond the nation-state, as is argued by Zagel and Lohmann (Chapter 10.1007/978-3-030-54618-2_6). We highlight a number of themes that emerged from the chapters in this Handbook that work toward such an agenda and conceptual focus.

First, there are clear differences between supra-national and international organizations when it comes to their normative views on family policy. In part, the different positions these organizations take with respect to family policy relates to their origins, mandate, and initial goals and geographic area of competence. Razavi demonstrates in Chapter 10.1007/978-3-030-54618-2_5 how there is not “one United Nations,” with a labor-centric focus in the ILO, a focus on children in UNICEF and UN Women centering key feminist concerns. UNIFEM (that later merged with DAW to become UN Women) focused on women’s economic rights, but operated on the assumption that various other family- and social policies were more relevant for middle- and high-income countries. Jenson (Chapter 10.1007/978-3-030-54618-2_3) shows how the OECD and the EU framed family policies in terms of their concern about too *low* fertility and social investment policies to foster growth, whereas the World Bank was concerned about too high fertility globally because of poverty and “limits to growth.” We point out here, that although the social investment perspective is now center stage in country-comparative work on social and family policy, it lacks clear analytical focus and misses out on key concepts in the family policy literature, such as unpaid work and gender inequality in paid and unpaid work (Cantillon & Van Lancker, 2013).

A second theme that emerged is that of the power of national governments. International Organizations are crucial to facilitate globalization and affect the interdependencies between countries, by being venues of mutual learning and cooperation, aligning collective interests, to spread norms and practices among member states, but also as a stage of confrontation of norms and influence. Policy ideas diffuse across nations through mechanisms that include competition, coercion, socialization/learning, and emulation (White in Chapter 10.1007/978-3-030-54618-2_4). Yet, the diffusion of ideas toward national policies is imperfect, norms and ideas are filtered through domestic mediating factors, including many factors affecting gender equality such as the number of women in parliament, veto players et cetera. In a globalized world, family policy is hardly formulated—let alone implemented—at a global or even supra-national level (Jenson in Chapter 10.1007/978-3-030-54618-2_3). Supra-national organizations have little hard power, although, for instance, the World Bank can use soft power by providing conditional loans. Even in the European Union (EU), where member states have delegated some of their legislative power, top-down influence remains opaque when it comes to family policy—although for example the 2019 Directive on Work-Life Balance suggests some change in this regard. Globalization notwithstanding, the national (or federal) level of family policy making remains key to understand family policies. Showing evidence that the multilevel policy levels act as communicating vessels, Parolin and Daiger von Gleichen report in Chapter 10.1007/978-3-030-54618-2_18 that in the United States federal-level policies compensated for state-level changes support for families. Although organizations play an important role in setting the “ final availability” of family policies to their workers (more on which below), Chung argues in Chapter 10.1007/978-3-030-54618-2_21 that the national context in terms of attitudes and policies set the limits and expectation in which organizations and their managers can operate. A similar adaptation to national-level conditions has also been identified in Bardoel’s Chapter 10.1007/978-3-030-54618-2_23 on multinational enterprises. As enterprises in rich countries became more active in developing countries through acquisitions and mergers, local managers had to adapt their central human resource policy to the local situation.

Thirdly, regional differences and decentralization processes were shown to matter greatly for family policies as well—despite the strong position of the nation-state discussed above. In Chapter 10.1007/978-3-030-54618-2_17, Engeman, shows how in the United States, state-level and even city-level developments innovate paid family leave policies with more entitlements than the federal government provides. As a form of bottom-up policy learning, these sub-national policy developments may provide leverage and hope to those who advocate national-level policy adoption. Nonetheless, as long as states have leeway in the way federal grants are spent, as in the case of *Temporary Assistance for Needy Families* (TANF), for instance, income protection for families can be jeopardized because some states pursue other objectives (such as marriage promotion). Great diversity in federalist context is not necessarily for the better for families, argue Parolin and Daiger von Gleichen in Chapter 10.1007/978-3-030-54618-2_18. Indeed, local implementations of (family) policy can differ widely from the national/federal policies, and this variation has been shown to have consequences for both the use and the outcomes of different policies (see Chapter 10.1007/978-3-030-54618-2_17 by Engeman, Chapter 10.1007/978-3-030-54618-2_18 by Parolin and Daiger von Gleichen, and Chapter 10.1007/978-3-030-54618-2_20 by Emery). Moreover, as argued by Schober in Chapter 10.1007/978-3-030-54618-2_19, even if policies are implemented in the same way, regional differences in norms or economic conditions may result in sub-national variation in the use and outcomes of these policies. This challenges some cross-national analyses, as well as provides interesting insights in the development of family policy over time with a focus on sub-national actors. As discussed by Engeman in Chapter 10.1007/978-3-030-54618-2_17, even though it may seem that the window for paid leave in the United States has closed, the policy developments at the state-level may provide an impetus to “breaking the liberal-market mold.”

Fourth, a global research agenda should avoid or at least be aware of Western assumptions (Bardoel in Chapter 10.1007/978-3-030-54618-2_23) and go beyond the heteronormative family idea, taking due account of families that do not adhere directly to prevailing norms (Evertsson, Jaspers, & Moberg in Chapter 10.1007/978-3-030-54618-2_16).

Finally, understanding family policy in this multilevel context poses incredible demands to data. Data infrastructures require major investments and long-term commitments, for which funding seems increasingly scarce (e.g., LIS, 2020; Nelson et al., 2020; Scruggs, 2013). Not only is greater investment in the quality, availability, and comparability of current databases imperative, making further progress requires the development of institutional data infrastructures along the lines developed by Sirén, Doctrinal, Van Lancker, and Nieuwenhuis in Chapter 10.1007/978-3-030-54618-2_24. Moreover, this work should not stop at the national level as is typically the case. Similar indicators at the sub-national and organizational levels would greatly further the research field as well. In that context, Chung calls in Chapter 10.1007/978-3-030-54618-2_21 for more research to genuinely gauge how company-level policies are shaped in the context of national and sub-national policies, and to what extent they are a reaction to existing policies, or are used as a tool to improve productivity rather than to foster work-life balance of workers. This is in particular a plea for gathering more cross-national comparative data on companies of different sizes in different sectors (see also the data presented by Begall and Van der Lippe in Chapter 10.1007/978-3-030-54618-2_22), so that a more integrative picture can be sketched of how international, national, regional, and company-level policies interact with one another, shaping actual work/family decisions and opportunities for families from different socio-economic backgrounds.

##  Austerity and Marketization

Although large parts of the (industrialized) world in Europe, the OECD and beyond have seen austerity and welfare state retrenchment (Beckfield, 2019; Taylor-Gooby, Leruth, & Chung, 2017), there seems to be no clear evidence that family policies were affected (e.g., Adema, Clarke, & Thévenon in Chapter 10.1007/978-3-030-54618-2_9). The European Union, for instance, seeks to expand paid parental leave to both parents, and paid family leave has become more common in some US states or cities (Engeman in Chapter 10.1007/978-3-030-54618-2_17). A challenge is to understand exactly why core family policies have expanded while other areas of policy were retrenched in many countries. Specific countries can of course show exceptions to this overall pattern, such as related to the introduction of the Universal Credit in the United Kingdom (Millar & Bennett, 2017). Parolin and Daiger von Gleichen show in Chapter 10.1007/978-3-030-54618-2_18 that state-level redistributive programs such as Temporary Assistance for Needy Families (TANF) have become less generous in a number of states, but this has (partially) been compensated by federal programs such as the Supplemental Nutrition Assistance Program (SNAP).

Two insights into this challenge arise from the chapters in this Handbook. First, it is worth noting in this context that supra-national organizations as the OECD and the EU changed their perspective from “family policy as a burden” in the 1980s to “family policies as a precondition for growth” (Chapter 10.1007/978-3-030-54618-2_3 by Jenson). Moreover, not all family policies were treated the same. Under the social investment paradigm in European countries, as well as the perspective on *active* social policy more generally, family policies that enable employment and foster human capital are front and center. Policies providing income protection such as family benefits are less popular, and were more subject to cuts under austerity and welfare retrenchment (see also below).

Second, the mode of family policy provision did change, and future research could pay more attention to how marketization is gaining more prominence. Razavi details in Chapter 10.1007/978-3-030-54618-2_5 how, for instance, the ILO has tried to maintain a high-road perspective on the public provision of care and regulation of care-sector jobs, while the UN sought more private sector solutions. Razavi identifies this as a risk, for “history tells us that market-based solutions are unlikely to provide the kind of universal social and family policies that can reign in gender, class and other intersecting inequalities.” In Chapter 10.1007/978-3-030-54618-2_8, Vandenbroeck raises similar concerns about marketization and demand-side financing of childcare provision, as it is associated with lower quality of service provision. Marketization tends to come with budget cuts on staff. Emery shows in Chapter 10.1007/978-3-030-54618-2_20 that in the Netherlands (a country with demand-side financing of ECEC) 70% of childcare facilities are now privately run, coming from 30% in 2005. Dykstra and Djundeva analyze in Chapter 10.1007/978-3-030-54618-2_14 how marketization in care services for later life families resulted in dualization. Witnessing a shift away from residential care toward home care, this potentially puts pressure on family relations in ageing societies—and particularly among those who do not have the means to compensate for inadequate provision of care by purchasing additional care on the private market.

## Economic Inequality

Economic inequality has once again taken a center stage position in public and academic debates in recent years. And it is, and will continue to be, highly relevant for family policy as well. Although the employment rates of women have grown closer to those of men over the last decades, the trends toward gender equality have slowed or even stalled in a number of countries ( for the US, see England, Levine, & Mishel, 2020), the gender pay gap has not closed (Goldin, 2014), occupational segregation is persistent (Charles & Grusky, 2004), and the different work histories of women and men contribute to gender gaps in old-age poverty (Möhring, 2015, 2016) that although closing, still persist (Doctrinal & Nieuwenhuis, 2019). Levels of wealth inequality (Piketty, 2014) and income inequality (Milanović, 2016) are high and rising within countries, as well as, for instance, in the European Union as a whole (Beckfield, 2019), and trends in poverty in Europe and beyond are “disappointing” (Jenkins, 2020; Vandenbroucke & Vleminckx, 2011). These are not mere concerns of accounting, but they have real-life consequences: The life chances of children growing up in different family forms are considered to be “diverging” (at least in the US, see McLanahan, 2004), more unequal societies impair equality of opportunity (Corak, 2013) and economic growth (OECD, 2015), and growing up in poverty has severe consequences for later life chances—even in rich societies (Van Lancker & Vinck, 2019).

Although these forms of inequality are intrinsically linked, it is useful to distinguish between regional, horizontal and vertical inequality. The distinction between horizontal and vertical inequality introduced in Chapter 10.1007/978-3-030-54618-2_3 by Jenson and Chapter 10.1007/978-3-030-54618-2_25 by Nieuwenhuis is relevant here to organize some of the key lessons learned. *Horizontal* inequality refers to differences between groups (such as men and women, parents and people without children, or single-parent families and two-parent families), *vertical* inequality refers of the overall differences between households (such as income inequality or poverty rates). Reminiscing the lessons learned in the previous section on the level of policy implementation, when considering how family policies may address horizontal and/or vertical inequalities it should first be recognized that there are also vast geographic inequalities in the availability of family policies.

### Regional Inequality

Regarding the geographic inequalities, the overview of family policy development in Chapter 10.1007/978-3-030-54618-2_10 by Filgueira and Rossell underlined the vast inequality across the globe in the fiscal capacity to implement family policies (and hence in the effectiveness of policies). In this context it is relevant to note that the majority of the world’s poor (defined in absolute income terms) no longer live in low-income countries but in fact live in countries that by now have evolved into middle-income countries (and within these countries overrepresented in specific, rural regions) (Sumner, 2016). These countries have—at least in theory—some budget to spend on family policies, which opens the question what family policies can mean for the global poor. Indeed, most countries provide rights and entitlements to at least some form of family policy (Heymann & Earle, 2010). Yet, implementation of, for instance, paid maternity leave and conditional cash transfers can be lacking with respect to coverage, eligibility criteria, entitlements, and non-take-up (see Chapter 10.1007/978-3-030-54618-2_10 by Filgueira and Rossel).

Chapter 10.1007/978-3-030-54618-2_18 by Parolin and Daiger von Gleichen demonstrated inequality in family policy availability, but then with regards to levels and take-up of family policies across states in the US. While living in the same country under the same federal state, this means that similar families have less/more access to better/poorer policies in terms of income, time and services because of the place where they (happen to) live. Schober in Chapter 10.1007/978-3-030-54618-2_19 shows similar evidence with regards to ECEC in Germany. So not only inequality across countries or between families within countries matters, but also between regions and states. Even beyond federalist states sub-national variation is relevant. Emery in Chapter 10.1007/978-3-030-54618-2_20 shows for the Netherlands that proximity of childcare facilities near one’s place of living determines women’s labor market opportunities. If childcare places are concentrated in well-off neighborhoods, as is discussed by Vandenbroeck in Chapter 10.1007/978-3-030-54618-2_8, this creates new barriers to employment. National policy frameworks with affordable care can still play out very differently across regions, so that once again where you live partly determines your opportunities.

###  Horizontal Inequality

Family policies have played a central role in reducing economic inequality between groups that include women and men, and single-parent and two-parent households. Yet, although there is a fair amount of consensus on the overall effects of paid leave and childcare on improving gender equality, little remains of this consensus when it comes to the impact of these family policies on class inequality. As Hook and Li synthesize the literature in Chapter 10.1007/978-3-030-54618-2_11, childcare increases the employment of women at any level of education, but the question remains who benefits most. Some studies find that the lower educated benefit more from the provision of childcare, others find the opposite. Clearly, such findings are closely connected to—and have implications for— vertical inequality, as discussed below. For paid leave there is a similar lack of consensus in the literature. How the intersection between gender and class plays out in relation to family policy, may very well depend on additional factors. Hook and Li suggest the type of (coordinated or liberal) market economy, cultural norms, or the overall level of income inequality. Along similar lines, in Chapter 10.1007/978-3-030-54618-2_7 Javornik and Yerkes call for analyses of the interplay between different types of family policies, and with other institutional and otherwise contextual conditions. Indeed, numerous outcomes envisaged with family policies can and are also achieved with a broader set of welfare state policies such as minimum income protection, unemployment benefits, housing benefits, labor market regulations, and overall redistribution (Alm, Nelson, & Nieuwenhuis, 2020; Bradshaw, Keung, & Chzhen, 2018; Horemans & Marx, 2018; Verbist, Diris, & Vandenbroucke, 2020).

With respect to class inequality in family policy outcomes, it has often been reported that higher-educated and higher-income parents are more likely to use formal childcare. Moreover, there is also evidence—as summarized in Schober’s Chapter 10.1007/978-3-030-54618-2_19—that shows that higher-educated and lower educated parents have different *types* of preferences regarding childcare. When selecting a childcare center, higher-educated parents tended to care more about the quality of the care on offer and the pedagogical curriculum, compared to lower educated parents. Native-German parents were less likely to choose childcare centers in which many children from migrant parents were enrolled. The implication of such findings is that childcare policies may not only reduce, but also perpetuate existing socio-economic inequality between groups of parents.

The perpetuation of inequality can also be seen at the company level. Family-friendly policies and flexible working arrangements are not available to all workers to the same extent. Usually high-skilled workers and higher-status jobs have more access to these company policies. Access is shaped by an individual’s real bargaining power (see Begall and Van der Lippe in Chapter 10.1007/978-3-030-54618-2_22). Moreover, there is also country-level inequality involved, since there is evidence provided by Chung in Chapter 10.1007/978-3-030-54618-2_21 that there is a positive relationship between family policies at the national or sub-national level and the company-level: companies tend to provide more generous policies in countries with more generous family policies. Linking regional and horizontal forms of inequality, it becomes clear that families living in countries with few family policies in place, or family policies disincentivizing women’s employment and preserving traditional gender norms in work and care, are facing a double jeopardy.

###  Vertical Inequality

A continuing challenge for family policies in OECD countries is to reach those families most in financial need, in order to play a more important role in the reduction of vertical economic inequality—including poverty. These goals and outcomes are clear at various levels of family policy making, as, for instance, demonstrated in the conceptual work on child income protection by Daly in Chapter 10.1007/978-3-030-54618-2_2, at the supra-national level as charted by Jenson in Chapter 10.1007/978-3-030-54618-2_3, the national level in Chapter 10.1007/978-3-030-54618-2_9 by Adema, Clarke and Thévenon and Chapter 10.1007/978-3-030-54618-2_13 by Maldonado and Nieuwenhuis, and the sub-national level of the state as demonstrated by Parolin and Daiger von Gleichen in Chapter 10.1007/978-3-030-54618-2_18.

Family policies indeed have the potential to reduce (child) poverty, but their effectiveness will be reduced if these family policies are only available in some sub-national areas in a country, are only used by highly-educated or high-income parents, or when companies or managers provide access to family-related policies only if they expect this to benefit the productivity of the workers—along the lines described above. This is where the link between horizontal and vertical forms of inequality in terms of family policy outcomes becomes apparent. This link was further developed by Nieuwenhuis in Chapter 10.1007/978-3-030-54618-2_25, arguing that there is some evidence that paid leave and childcare policies facilitate women’s employment to such extent that this helps reduce vertical economic inequality among the households of couples. Yet, to fully understand how family policies may affect vertical income inequality, it was argued, requires not only to consider the income effects of using family policies but also to consider who uses family policies and with whom they live. Although the argument focused on national-level policies only, in the context of this handbook this immediately brings into focus to other levels of policy making. From this perspective, sub-national variation in the availability of family policies may not only affect economic differences within regions (or states) but also between. Who uses family policies is also determined by the “ final availability” determined by organizations. This can, for instance, relate to organizations restricting access for (some) workers to flexible working arrangements, to workers avoiding to use their legal entitlements in anticipation of repercussions, or companies providing workers with more generous family policy arrangements than publicly provided or mandated. The extent to which organizations provide or limit access to worker’s access to such policies, and whether they do so following a socio-economic gradient, may be an important additional mechanism shaping vertical economic inequality.

## Changing Family Relations

The diversity of family forms vastly exceeds that of the number of ideal-typical, model family types that family policy makers implicitly or explicitly have in mind. Family configurations consist of a wide range of interdependencies among family members, that need not be based on kinship, need not live in the same home, and that are subject to change over time (Widmer, 2010). The ideal-typical “nuclear family,” consisting of a married husband and wife with dependent children, by no means describes the reality of a majority of families ( UN Women, 2019). The capability approach, as developed in Chapter 10.1007/978-3-030-54618-2_7 by Javornik and Yerkes and Chapter 10.1007/978-3-030-54618-2_19 by Schober, provides a framework that is inherently sensitive to a diversity of life-courses and to family diversity. In its recognition of individual’s agency as socially embedded, it helps explain how family policies can have widely different consequences for different individuals or families. The challenge for family policy makers and scholars alike is how to implement family policies that support this wide range of families. This resolves around a number of issues, including the definition of family types, whether or not different family types require specific policies, and solidarity among family types.

The importance of definitions was raised prominently by Evertsson, Jaspers and Moberg, who in Chapter 10.1007/978-3-030-54618-2_16 introduce the concept of parentalization to address the issues of who can become a parent and how, and who can share in the care of the child. Applied to female same-sex couples , this chapter highlights the importance of the definition of concepts as “parents.” Even in countries that are typically considered to be rather liberal, laws, policies and ideas about who can become a parent (in a variety of interpretations of “parent” that include the social, the legal, and the biological) lag behind the reality of couples who want to become a parent and want to be able to take upon themselves all the responsibilities that come with parenthood. Family policies enacted in law can deliberately exclude certain types of families or favor one particular type of family. In reality, many parents who do not adhere to the gender norm of the heteronormative family are less or not entitled to parental leave schemes or face steep barriers to become parents in the first place. A number of developments, including extending IVF and adoptive rights to same-sex couples and expanding the number of legal parents a child can have, work toward more inclusive notions of parenthood. Here, it is to an important extent the changing of the definition of a “parent” that includes more people in the reproductive, social, and family rights that were already enjoyed by others. In the area of care (leave) for frail and elderly family members, similar debates arise regarding who is considered “family” (Ivanova & Dykstra, 2015).

The issue whether there is a need for group-specific policies was addressed in the two chapters on single parents. Chapter 10.1007/978-3-030-54618-2_12 by Skinner and Hakovirta discuss child support policies that were designed to specifically address the needs of (children growing up with) separated parents. Child support arrangements often represent long-term commitments, as payments are due typically until children reach adult age. During this time, a lot can change in the lives of both separated parents, in terms of, for instance, income and employment, re-partnering, and having more children (with other partners). Over time, family relations have been changing away from the traditional breadwinner model. An important challenge for (the administration of) child support systems is to adapt to these new situations—sometimes this challenge seems nigh insurmountable (Meyer, Skinner, & Davidson, 2011). They show that only in a minority of countries child support systems adapted to reflect changes in maternal labor market participation, e.g., by taking account of mother’s earnings in calculating child support amounts, or acknowledge the role of fathers in childcare. In addition, as the chapter concludes, the goals of promoting gender equality and of child income support can be competing. This analysis is complemented in Chapter 10.1007/978-3-030-54618-2_13 by Maldonado and Nieuwenhuis with the argument that single parents often benefit from policies that are aimed at all families with children—not just for single parents. So, instead of child support, this chapter examined whether single parents benefit just as well as two-parent families from child income supports, childcare and paid parental leave. The results for family benefits were unequivocal: when it comes to poverty reduction, single parents benefit more from family benefits than two-parent families. Other work argued that the poverty reduction associated with family benefits can exceed those of child support (Nieuwenhuis & Maldonado, 2018). With respect to parental leave and childcare, the results are less clear-cut: single parents receive similar or slightly higher payments during parental leave and pay similar or slightly lower fees for childcare. However, expressed relative to their household income the replacement rate of parental leave is lower, and the childcare fees higher, for single-parent families compared to two-parent families. While single parents are in pressing need of reconciliation policies if they want to work, those policies are less affordable for them.

The third issue, solidarity among family types, can best be illustrated with chapters on care for elderly and frail family members. With increasing longevity and population ageing, it is increasingly important to also consider care relations between (adult) children and their elderly parents, and policies that can support this form of care as well. In Chapter 10.1007/978-3-030-54618-2_14, Dykstra and Djundeva chart such policies for long-term care (LTC), and it becomes clear that such policies can comprise of a combination of providing care directly to the elderly (elderly homes, home care) and providing support for the family care giver (e.g., in the form of leave, or awarding pension credits to care givers). Nevertheless, despite the coordinating efforts of the European Commission in the *Pillar of Social Rights*, many welfare states (in particular in Southern and Eastern Europe) fail to ensure adequate care for their elderly and frail citizens. Witnessing a shift away from residential care toward home care, this puts pressure on family relations in ageing societies. In-kind policies such as residential care come with different trade-offs compared to cash-for-care schemes, for instance, with respect to refamilization and gender equality (in both care and work). Dykstra and Djundeva call in Chapter 10.1007/978-3-030-54618-2_14 for policy evaluations that “*cut across* policy domains”. This issue of cross-domain evaluations relates to the starting point of Birnbaum, Ferrarini, Nelson, and Palme (2017), who showed that welfare states providing similar levels of financial support to children, working-age people, and the elderly tended to have higher levels of overall support compared to countries that focused their financial support on only one age group. Such findings are important to advocates for family policy as well, as shown by Engeman in Chapter 10.1007/978-3-030-54618-2_17. Advocates for paid family leave in the United States made sure not to frame their policy proposals as aimed at care for a specific group, or to support women, but instead anticipated greater support if their proposals were formulated in a gender-neutral and universal frame to provide care for all family members—irrespective of their age.

## Gender Revolution: Adapting to Women’s Empowered Roles?

 Developments across OECD countries in terms of family policies helped reduce gender inequality, and improved fertility rates (or rather, perhaps, slowed the decline in fertility). Although the initial rise in female labor force participation was linked to fertility decline and relationship dissolutions, the stagnation or even reversal of these trends was linked to societies—and in particular men—adapting to the norm that women are highly-educated and have empowered roles (Esping-Andersen, 2016; Van Bavel, Schwartz, & Esteve, 2018). Nonetheless, the care for children, as well as for elderly parents (and other family members) is predominantly shouldered by women, which also has important implications for their working lives and later their own retirement income (Dykstra and Djundeva in Chapter 10.1007/978-3-030-54618-2_14). It is a challenge to design and provide family policies that adequately promote gender equality in terms of labor, care, and leisure.

 Adult worker models that are often used to theorize comparative family policy research, usually lack a focus on gender equality in the labor market as well as in unpaid work—as detailed by Zagel and Lohmann in Chapter 10.1007/978-3-030-54618-2_6. To be able to think about changing gender and class relations simultaneously, it is important to further develop concepts and theories to examine asymmetrical and heterogeneous policy effects (also see Chapter 10.1007/978-3-030-54618-2_11 by Hook and Li).

Family policies, and welfare states more generally, have been struggling to adapt to changing gender relations and women’s empowered roles, which was detailed in numerous chapters of this handbook. We outline three challenges. In many countries paid parental leave for fathers continues to lag far behind parental leave provisions to mothers (Adema, Clarke, and Thévenon in Chapter 10.1007/978-3-030-54618-2_9), but welfare states are in fact adapting their parental leave policies to also allow—and encourage—fathers to take parental leave. Fathers’ taking of leave can in itself be considered as a form of gender equality, and it may foster other forms of gender equality that reduce income differences within households, and may represent more gender-equal role models for their children. Still, in Chapter 10.1007/978-3-030-54618-2_15 Bartova and Keizer show vast differences between countries, in terms of the duration of leave that fathers can take, the level of wage replacement, as well as in which way leave is provided to fathers (e.g., non-transferable leave, or individual entitlements or family entitlements). Individual, non-transferable leave for fathers was found to be the most effective policy design to encourage fathers to take parental leave.

A second challenge for welfare states to adapt to women’s changing roles is also clear with respect to child support systems. Skinner and Hakovirta show in Chapter 10.1007/978-3-030-54618-2_12 that child support systems effective in reducing child poverty are not always the ones in which gender equality is taken on board—as was discussed in more detail in the previous section. In systems which are based on a male breadwinner model, the income of the mother is usually not taken into account for calculating support amounts. This runs against ambitions to promote gender equality, while these systems are more effective in reducing poverty.

Also related to single parents, and not covered in this handbook, is how the rise of shared residence also represents changing gender relations. Shared residence is the practice that children continue to live about equal amounts of time with both their parents after they separated (Fransson, Låftman, Östberg, & Bergström, 2018). To the extent that separated fathers and mothers are both actively involved in the care for their children challenges the notion that most single parents are mothers (Nieuwenhuis, 2020). Shared residence seems to be on the rise in a number of European countries, and there is evidence to suggest that this benefits the well-being of these children (Baude, Pearson, & Drapeau, 2016; Nielsen, 2014), although it should be acknowledged that parents doing shared residence tend to be a selective, rather well-resourced group and that the shared residence living arrangement is relatively unstable (Poortman & Van Gaalen, 2017). Shared residence is to be understood as relationships between (individuals in) multiple households, and therefore typically not captured well in large-scale surveys—including those used to create the indicators that policy makers rely on. Yet, it is important to better understand the driving forces and outcomes of shared residence, and which policies might promote it and stimulate positive outcomes.

A third (set of) challenge(s) pertain to the observation that welfare states adapting their family policies to changing gender roles may not be enough. The role employers and organizations can play in this respect, is not yet fully understood. On the one hand, Goldin (2014, p. 1091) argued that employers have a central role to play in the “last chapter” of gender convergence in pay: a sizeable part of the gender pay gap in high-paying occupations in the United States is related to working conditions that favor very long work hours and are inflexible with respect to when and where the hours are worked. On the other hand, based on data from multiple countries, Chung discusses in Chapter 10.1007/978-3-030-54618-2_21 how family-friendly arrangements and flexible working at the company level might actually contribute to stronger patterns of inequality in work and care. In a flexible environment, men are more likely to increase their overtime hours while women are more likely to increase time spent on household chores and care work. These outcomes of workplace flexibility are shaped by gendered norms and expectations attached to gender (as well as to level of education, as shown by Begall & Van der Lippe in Chapter 10.1007/978-3-030-54618-2_22). The flip side of this, of course, is that in countries with more egalitarian gender norms, the effect of flexibility will be different as well. This goes to show that gender inequalities are likely to be reproduced at the organization level if dominant country norms are not challenged. At the same time, flexible working and family-friendly working arrangements might reduce gender inequality in the labor market. When women are able to retain control over their working time, this might reduce the need for transitioning into part-time jobs, which come with wage and career penalties, and the gender wage gap tends to be smaller in companies with more family-friendly policies, in particular flexible working arrangements. Multinational enterprises often operate in contexts with very different gender norms and expectations about work and family, resulting in tensions between (typically) the Western notion of work-life balance and national norms, practices, and challenges. Bardoel (in Chapter 10.1007/978-3-030-54618-2_23) presents ample examples of the very different types of support human resource managers provide to their workers in different parts of the world. Being able to adjust to these very different needs in the context of an enterprise that operates in multiple countries requires the recognition and understanding of tensions. Such tensions can be distinguished along dimensions that include strategic vs. operational concerns, centralization vs. decentralization, or institutional versus contextual awareness. The need to addressing such tensions effectively and based on evidence, raises new questions of human resource management in multinational enterprises.

## The Next Decade of Research: Family Policy in Extraordinary Times

Much of the research on family policies examines how and why family policies change over time, and how changing family policies (or differences in policies across countries, regions, or organizations) are related to a variety of outcomes for a variety of families. Much less, however, is known about the development and effectiveness of family policies when societies undergo rapid change. What does the evidence generated in *ordinary times* tell us about the role of family policies in *extraordinary times*? At the time of writing this conclusion, early May 2020, the rapid spread of the COVID-19 pandemic has had a massive impact on societies across the globe. It is too early to write anything definitive, but too late not to write anything. Countries almost universally shut down large parts of their economies to reduce the spread of the pandemic and to prevent healthcare systems to become overwhelmed. Marked increases of all-cause mortality were visible almost everywhere, in particular among the elderly (EuroMOMO, 2020). Quite possibly, at the time of reading, it has become clear that this was only the beginning. But the current crisis reminds us of the recurring nature of crises: the 2008 financial and economic crisis (“Great Recession”), the 2015 wave of migration in European countries, partly caused by violent conflicts in Syria, Iraq, Afghanistan and other countries, and concerns about the consequences of climate change are all examples of crises that can alter the course of societies. The very Swedish model was motivated by concerns about emigration and low fertility, in a book famously titled “Crisis in the population question” (Myrdal & Myrdal, 1934). Although we never know when crises hit, we can prepare for the eventual next one. Yet, very little seems to be known about the fundamental question of whether and how family policies function in times of societal upheaval, and for whom. The five challenges we put forward in the previous section can give us direction on how exogenous shocks, crises, and in particular their economic consequences, raise pertinent questions for the next decade of family policy research.

The evidence collected in this handbook suggests that supra-national organizations have had a limited direct influence on the making of family policy, although OECD, ILO and UN Women all provide analyses and recommendations, and the EU has had tangible impact on member states’ family policies. While budgetary restrictions in the Eurozone have been associated with austerity (Beckfield, 2019), in times of societal upheaval and economic crisis, organizations like the EU can provide financial support to member states to keep their economies afloat. The Great Recession of 2008, for instance, was followed by European coordinated stimulus policies to absorb the shock and to avoid the Eurozone from collapsing in the short-term. In the longer-term, however, a straightjacket of fiscal austerity was imposed on several countries by the European Commission, the European Central Bank and the International Monetary Fund. This led to austerity measures being implemented in many member states, affecting some family policies as well. Most affected were cash transfers such as family and child allowances. Following the crisis, in countries such as Greece, the Netherlands, the UK, Hungary but also Finland, cuts in benefits were implemented under fiscal consolidation measures including a freeze of benefit levels, tighter eligibility conditions, the abolition of tax breaks, or actual reductions of child benefits (Richardson, 2010; Thévenon, Adema, & Ali, 2014). The effect of the 2008 crisis in terms of the labor market as well as the austerity measures put in place in the aftermath affected children particularly hard (Cantillon, Chzhen, Handa, & Nolan, 2017; Chzhen, 2017). However, as family policies are still the prerogative of EU member states, little is known about how a more coordinated approach toward family policies would affect their capacity to cope with shocks of various kinds. While family transfers were subjected to cuts, the effect on leaves and particularly childcare services was different. Usually, pre-crisis reforms were carried out as planned, and childcare is a policy area to which governments increasingly devoted public resources (Van Lancker & Ghysels, 2014). However, here too, the challenges for research we identified are relevant. An increase in spending is meaningless as such; what matters is how public budgets are spent. Concern has been raised that the 2008 economic crisis has put additional pressure on public budgets, accelerating a process of marketization of public provision of care services, including long-term care and childcare services. This raises the question what the consequences might be for the generational conflict (Birnbaum et al., 2017). For instance, in the face of budget constraints, how will public care for children be prioritized relative to care for people later in life? Marketization of public service provision was linked to a dualization between those who can afford services and who cannot, ranging from the provision of childcare to elderly care. Yet, little is known about whether the crisis exacerbated existing processes of dualization to a larger extent in marketized systems of provision compared to public provision of (care) services.

With respect to decentralization, for instance, municipalities providing public services to families, a concern is whether regional variation in the provision of services as well as in the degree to which municipalities are affected by crises, translates into—or exacerbates—regional inequality with respect to access to services and how well people are protected by these services.

A primary concern regarding (the economic consequences of) crises is rising inequality. Social policies have been described as *automatic stabilizers* in times of economic downturn. This has most notably been described in the context of unemployment insurance, that automatically stabilizes incomes of workers and their families (at least to some degree and typically for a limited period of time) even when unemployment rises rapidly. The benefit is not only to the families receiving the benefits, but also to the economy at large because unemployment benefits stabilize purchasing power and demand for goods, thus helping to prevent further collapse of companies. Family leave policies, including the ability to take time off from work for own illness or to care for a family member, have been described in a similar manner (Boushey, 2016). For instance, at the time of writing, many countries including Slovenia, Belgium, Finland and Poland had implemented or expanded leave policies for parents who cared for young children during the COVID-19 lockdown measures. It remains to be seen whether these expansions will be temporary, or lead to structural adjustments of leave policies in these countries. Child benefits are not an automatic stabilizer per se (as they are always provided, not only in times of economic turmoil), but are well-known to be highly effective to help reduce poverty in large parts of the world and among a wide range of families with children (see, for instance, Chapter 10.1007/978-3-030-54618-2_9 by Adema, Clarke, & Thévenon, Chapter 10.1007/978-3-030-54618-2_10 by Filgueira & Rossel, and Chapter 10.1007/978-3-030-54618-2_13 by Maldonado and Nieuwenhuis). We have yet to learn how automatic stabilizing mechanisms provided by family policies in combination with other income protection policies will hold up in the face of (health- and economic) crisis. It will be an important question to examine how their presence may help families as well as societies endure the pandemic, equalizing risks, and hasten recovery.

Greater disruptions might be expected when it comes to (public or private) service provision. This includes—but is certainly not limited to—childcare, schooling and care for elderly. In relation to distancing measures, many childcare facilities and schools have been closed. Otherwise, services provided by the company (see Chapter 10.1007/978-3-030-54618-2_21 by Chung for examples and how common such company-level services are across European countries) are tied to employment, and workers lose access when they are unemployed or when the company goes bankrupt. More generally, it was found that in times of economic crisis, managers put more emphasis on whether the company might benefit from providing company-level services, rather than on the needs of the worker (Been, Den Dulk, & Van der Lippe, 2016).

Childcare and other forms of education serve an important function in equalizing development and equal opportunities in children. From research on long holidays we can learn about potential consequences of long closures of childcare facilities and schools (Campbell, Watson, & Watters, 2015), and the evidence strongly suggests that long closures will exacerbate inequality in the skills, knowledge and development of children with different socio-economic background. Moreover, schools also provide supervision on other aspects of well-being and (social) safety, and support in the form of school meals. As such, long closures of childcare and schools—or parents losing access to company provided care services—are a risk factor for heightened inequality (Van Lancker & Parolin, 2020). Family policy researchers should do their part examining to what extent such arising inequalities have been most effectively prevented—or remedied—by different modes of (for instance) childcare provision. Horizontal inequalities in terms of ageing and caring are also highly relevant to study in relation to crisis. The spread of COVID-19 infections is particularly dangerous for the elderly. How families live and care together, and to what extent welfare states provide adequate care for frail elderly may prove to be relevant factors to understand why and to what extent some countries are more severely affected by the pandemic than others. As such, how families and welfare states are able to absorb shocks is determined by how welfare states organize care relations.

From the perspective of family diversity , it should be recognized that some family forms might be in a better position to deal with (the consequences of) a crisis than others. Therefore, the importance of family policy may also vary. From the perspective of family policies, the potential consequences of a crisis are myriad. How will the closure of childcare facilities in response to a crisis, for instance, affect the challenge for single parents to combine work and family responsibilities? How are child support payments affected if parents lose their jobs and are no longer able to make the payment—how will this affect family relations? How do separated parents (re-)negotiate shared parenting arrangements during societal upheaval? How effective can care regimes for later-in-life families that rely on family members providing care operate when the financial or health situation of these family members changes rapidly? What will be the consequences of closing down (or no longer financially supporting) IVF centers—even if only temporary—for the ability of a wide range of families to become a parent? It should finally be recognized that family policies might not be enough for all families: lowered levels and stricter eligibility criteria in unemployment insurance benefits have rendered families without a second earner in the household into a new risk group for poverty (Alm et al., 2020). As such, the capacity of unemployment benefits to act as an automatic stabilizer as discussed above may be inadequate for different types of families in times of crisis.

Finally, it will remain to be seen—and extensively studied—what direction the gender revolution will take in times of crisis, and what role family policy can continue to play here. Women spend more time than men on care work, and when childcare services (or schools) are not available it is a real concern that this gender inequality increases. It has been shown that fathers who took parental leave are more involved with the care for their children later on (Duvander & Jans, 2009): it will be interesting to see whether this (change in) behavior persists in times of crisis. Evaluations of the ( austerity following) the 2008 financial crisis showed that particularly the public sector was hit by a reduced number of jobs and wage cuts. Since more women than men work in these sectors, they were more likely to be affected by these measures (Rubery, 2015). As it is an ongoing debate whether family policies reduce or increase class-based inequality, including occupational segregation, this raises the issue whether current constellations of family policies facilitate women’s activity in sectors that are more vulnerable during and after crisis and other forms of major societal change.

As policies to support families in terms of work, care, leisure, and incomes evolved and developed across the world, from companies, over cities and regions, to nation-states and supra-national and international organizations, a clear research agenda for the next decade emerges. Many societal challenges lie ahead of us, of which we identified five in this concluding chapter, and how family policies develop in the future will affect how these challenges unfold and affect families. In addition, we identified that there are clear gaps in our knowledge on how to adequately support ordinary families in extraordinary times. We sincerely hope our handbook will prove to be an anchor point, synthesizing what we know while contributing to the research agenda on what we need to know.
